# Two Surgical Cases of Thymic Tumor Requiring Resection and Reconstruction of the Superior Vena Cava and the Brachiocephalic Vein

**DOI:** 10.3400/avd.cr.25-00145

**Published:** 2026-03-27

**Authors:** Makoto Shirakawa, Hikari Kimura, Ryosuke Amitani, Kenji Suzuki, Yasuo Miyagi, Jiro Honda, Kento Suzuki, Takumi Sonokawa, Yuichiro Machida, Norihito Kawasaki, Jitsuo Usuda, Yosuke Ishii

**Affiliations:** 1 Cardiovascular Surgery, Nippon Medical School Hospital, Tokyo, Japan; 2 Thoracic Surgery, Nippon Medical School Hospital, Tokyo, Japan

**Keywords:** resection and reconstruction, major vessel, extracorporeal circulation

## Abstract

Surgery for thymic tumor involving major vessels remains challenging; however, complete surgical resection is recommended when the resected major vessels can be reconstructed safely. Two cases are described in this report. In Case 1, complete surgical resection followed by reconstruction using an autologous pericardial patch was achieved owing to the temporary bypass assist. In Case 2, complete surgical resection followed by reconstruction using an autologous pericardial roll was achieved owing to the cardiopulmonary bypass assist. Complete surgical resection of thymic tumor involving the major veins can be achieved safely with appropriate extracorporeal circulatory assist. Autologous pericardium is useful for venous reconstruction.

## Introduction

Complete surgical resection is crucial for thymic tumor; however, it can be challenging as it sometimes involves the major vessels.^1)^ Although thymic tumor involving the superior vena cava (SVC) and the brachiocephalic vein (BCV) has been traditionally considered unresectable, and surgery for such tumor has been challenging,^2)^ complete surgical resection is recently recommended when the resected major veins can be reconstructed safely, according to the concept of oncovascular surgery.^3,4)^ We report 2 surgical cases of thymic tumor involving the SVC and the bilateral BCVs.

## Case Report

### Case 1

**Fig. 1:**
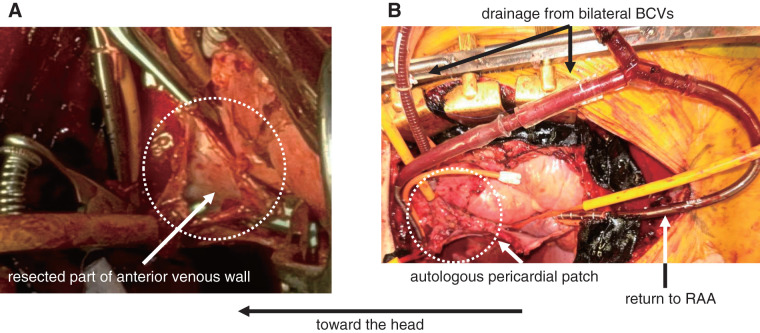
(**A**) Resection of thymic tumor with anterior venous wall of SVC and bilateral BCVs. (**B**) Venous reconstruction with autologous pericardium using temporary bypass assist. (**A**) The thymic tumor was resected with the anterior wall of the SVC and bilateral BCVs as indicated by the white dotted circle. (**B**) Temporary bypass was established by cannulating the bilateral BCVs for venous drainage and the RAA for venous return during tumor resection and venous reconstruction, as indicated by the arrow. The resected part of the venous wall was reconstructed using an autologous pericardial patch as indicated by the white dotted circle. BCV: brachiocephalic vein; RAA: right atrial appendage; SVC: superior vena cava

A 48-year-old male diagnosed with a thymic tumor underwent tumor resection by thoracic surgeons. Intraoperatively, the tumor was found to involve the anterior wall of the SVC and the bilateral BCVs. Therefore, cardiovascular surgeons planned to resect these venous walls and reconstruct them using a temporary bypass assist from the bilateral BCVs to the right atrial appendage (RAA). Median pericardiotomy was performed to expose the SVC and the RAA, and adequate fresh autologous pericardium was prepared for venous reconstruction. Temporary bypass was established by cannulating the bilateral BCVs for venous drainage and the RAA for venous return with an activated clotting time (ACT) maintained at >200 s using low-dose heparinization. The height of the temporary bypass was set low to ensure smooth venous return from the upper body. After clamping the bilateral BCVs and the SVC at the distal site of the azygous vein connection, the tumor was successfully resected along with the anterior venous walls with an adequate margin under well-controlled bleeding (**Fig. 1A**). The resected part of the SVC and the bilateral BCVs was reconstructed using a trimmed fresh autologous pericardial patch (**Fig. 1B**). The part of reconstruction was confirmed to be successful using direct ultrasonography. The duration of the temporary bypass was 56 min. The cerebral venous pressure did not change, and the cerebral regional oxygen saturation (rSO_2_), monitored using the O_3_ (Masimo, Irvine, CA, USA), was maintained at approximately 65% before and after clamping throughout the procedure. All cannulas used for the temporary bypass assist were then removed. The total blood loss during surgery was 212 mL. Neurological deficit was not detected, and the tracheal tube was removed on the 1st postoperative day. He was discharged on the 8th postoperative day without any complications. The final pathological diagnosis was type B3 thymoma (WHO classification), stage IIb (Masaoka-Kaga stage), and T2N0M0 (TNM pathological staging). Furthermore, complete surgical resection of the tumor with adequate margin was confirmed.

### Case 2

**Fig. 2:**
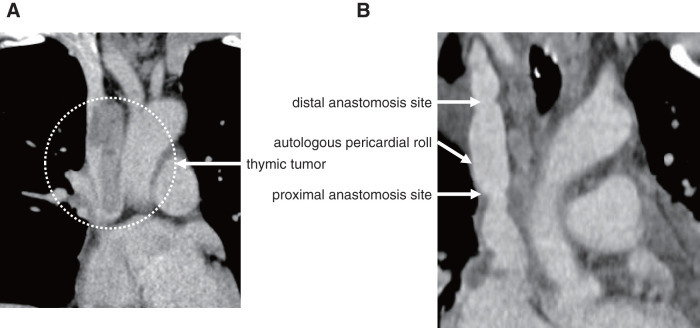
(**A**) Preoperative computed tomography findings. (**B**) Postoperative computed tomography findings. (**A**) The distorted thymic tumor invaded into the SVC and extended into the RAA, as indicated by the white dotted circle. (**B**) Computed tomography on the 7th postoperative day revealed good patency of the autologous pericardial roll. Both anastomoses of the autologous pericardial roll are indicated by white arrows. SVC: superior vena cava; RAA: right atrial appendage

**Fig. 3:**
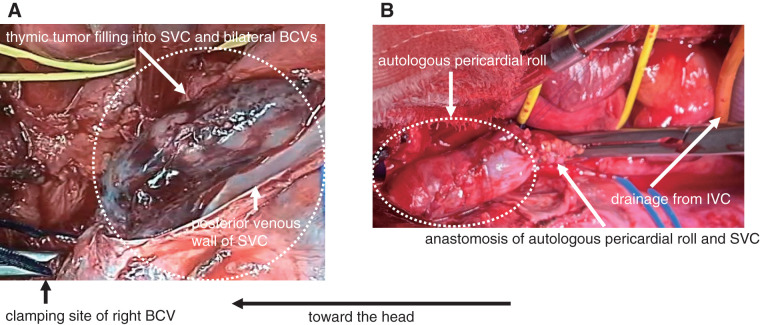
(**A**) Thymic tumor invading into SVC and bilateral BCVs. (**B**) Venous reconstruction with autologous pericardium using CPB assist. (**A**) Thymic tumor filling into the lumen of the SVC was confirmed by extension of the right atriotomy toward the SVC, as indicated by the white dotted circle. (**B**) The resected SVC and right BCV were reconstructed using an autologous pericardial roll, as indicated by the white dotted circle. BCV: brachiocephalic vein; CPB: cardiopulmonary bypass; IVC: inferior vena cava; SVC: superior vena cava

A 48-year-old female diagnosed with a thymic tumor invading into the SVC (extended to the RAA), the bilateral BCVs, and the upper lobe of the right lung underwent tumor resection by thoracic surgeons (**Fig. 2A**). Intraoperatively, the tumor was confirmed to have invaded into the SVC and the bilateral BCVs as indicated by preoperative computed tomography. Therefore, cardiovascular surgeons planned to resect these veins and reconstruct them using a cardiopulmonary bypass (CPB) assist. CPB was established by cannulating the right jugular vein and the inferior vena cava (IVC) for venous drainage and the right femoral artery for arterial inflow with an ACT maintained at >400 s using full heparinization. The left BCV was ligated after confirming the cerebral venous pressure via the left jugular vein under temporary clamping. Right atriotomy was performed after establishing total CPB circulation, revealing tumor extension into the SVC and the bilateral BCVs (**Fig. 3A**). The tumor, the SVC, and the bilateral BCVs were successfully resected en bloc with an adequate margin under well-controlled bleeding. The resected SVC and right BCV were reconstructed using a trimmed fresh autologous pericardial roll, which was constructed by rolling fresh autologous pericardium into a tubular shape corresponding to the length of the resected segment and the diameter of the SVC (**Fig. 3B**). The part of reconstruction was confirmed to be successful using needle direct pressure study. The CPB duration was 141 min. The bilateral cerebral venous pressures did not change, and the cerebral rSO_2_ was maintained at approximately 65%–70% before and after clamping throughout the procedure. All cannulas used for the CPB assist were then removed. En bloc complete surgical resection of the tumor, including the invaded major veins and the upper lobe of the right lung was achieved. The total blood loss was 1215 mL. Neurological deficit was not detected. The reconstructed part demonstrated satisfactory patency on the 7th postoperative day, as confirmed by computed tomography (**Fig. 2B**). Re-exploration was required on the 13th postoperative day for mediastinitis due to pyothorax caused by a right lung fistula, and she subsequently underwent continuous irrigation and negative pressure drainage under open sternum. As the infection became immediately well-controlled, sternal closure was successfully performed on the 22nd postoperative day. The tracheal tube was removed on the 36th postoperative day, and she was discharged on the 74th postoperative day after rehabilitation. The final pathological diagnosis was type B3 thymoma (WHO classification), stage IVb (Masaoka-Kaga stage), and T3NoM1b (TNM pathological staging). Furthermore, complete surgical resection of the tumor with adequate margin was confirmed.

## Discussion

The concept of oncovascular surgery has expanded the indications for aggressive complete surgical resection of thymic tumor involving the major vessels.^4)^ The complete surgical resection of the advanced thymic tumor significantly contributes to superior outcomes; notably, the 5-year survival rate of stage III and IV cases (Masaoka-Koga staging) was 92.9% in patients who achieved complete surgical resection compared with 35.6% in patients with inoperable tumor.^5)^ The recent Japanese guidelines recommend complete surgical resection of thymic tumor involving the major vessels when surgery can be performed safely;^3)^ however, standard surgical techniques have not been fully established. We described our approaches for the safe resection and reconstruction of the SVC and BCV in thymic tumor involving these veins.

First, it is essential to determine whether extracorporeal circulatory assist is required and which modality should be selected, balancing safety and invasiveness in oncovascular surgery. Previous studies have reported that simple clamping of the SVC for 30–60 min does not lead to life-threatening complications.^2,6,7)^ By contrast, simple clamping of the SVC, especially at the proximal site of the azygous vein connection, can lead to brain damage due to brain edema caused by increased cerebral venous pressure and hypotension due to reduced venous return from the upper body.^6)^ We established extracorporeal circulatory assist to prevent such complications in the 2 reported cases. In Case 1, temporary bypass assist from the bilateral BCVs to the RAA was established as the surgical procedure was expected to be simple, because tumor invasion was limited to the anterior wall of the SVC and the bilateral BCVs, allowing straightforward reconstruction with an autologous pericardial patch. By contrast, CPB assist was employed in Case 2 to ensure safety, as the extent of tumor invasion was unclear and reconstruction was assumed to be more complex. CPB assist provides safer and more reliable extracorporeal circulation; however, temporary bypass assist is a less invasive alternative.^8)^ The safe use of a temporary bypass requires careful consideration of factors such as the length and height of the bypass circuit. Because venous return through the temporary bypass depends on the venous pressure gradient created by clamping, the length of the bypass circuit should be kept as short as possible, and its height should be set low to ensure smooth venous return from the upper body.

Second, reliable monitoring of cerebral venous pressure is essential for detecting cerebral congestion. In our 2 cases, cerebral rSO_2_ monitoring was added to venous pressure monitoring. Cerebral venous pressure is generally regarded as the most reliable parameter, with a threshold of 30 cmH_2_O considered feasible and safe.^9)^ Although the reliability of cerebral rSO_2_ monitoring remains controversial, it has been reported that the elevated cerebral venous pressure and the lower cerebral tissue oxygenation are correlated.^10)^ Furthermore, cerebral rSO_2_ monitoring may help prevent neurological dysfunction.^11)^ Therefore, combining cerebral rSO_2_ monitoring with cerebral venous pressure monitoring may be clinically meaningful. Additionally, maintaining cerebral rSO_2_ within the safe range (absolute rSO_2_ > 55% or a decrease of rSO_2_ < 20% from baseline^12)^) during SVC clamping provides reassurance to surgeons.

Finally, fresh autologous pericardium was used for reconstruction of the resected venous walls in our 2 cases. Several materials have been proposed for such reconstruction, including autologous pericardium, heterologous pericardial sheet, and prosthetic vascular graft.^1,2,7)^ Autologous pericardium is preferred to other materials due to its lower risk of infection and thrombosis.^1)^ In Case 2, although mediastinitis occurred secondary pyothorax due to a right lung fistula, continuous irrigation and negative-pressure drainage for 9 days could control the infection well. The use of autologous tissue may prevent critical infection such as graft infection, and facilitate effective infection control. Postoperative anticoagulant therapy is generally unnecessary after reconstruction with autologous pericardium but requires when prosthetic vascular graft is used.^1,13)^ In our cases, anticoagulation was not administered in Case 2 because of postoperative complication, whereas low-dose anticoagulation (oral edoxaban 30 mg daily) was prescribed for 1 month in Case 1. Moreover, the autologous pericardium is flexible and can be easily shaped as needed.^1)^ Therefore, it is an ideal material and should be considered the first choice for reconstruction of the major venous walls or veins.^1)^

## Conclusion

We report 2 cases of thymic tumor involving the major veins in which safe resection and reconstruction of the SVC and the BCV were achieved using appropriate extracorporeal circulatory assist. Establishment of extracorporeal circulation was straightforward for cardiovascular surgeons and helped prevent life-threatening complications. Additionally, autologous pericardium proved to be an effective material for major venous reconstruction. Surgery based on the concept of oncovascular surgery may increase the curability of tumor involving the major vessels.

## Declarations

### Data availability

Data availability is not applicable.
